# A Perspective on Interactive Theorem Provers in Physics

**DOI:** 10.1002/advs.202517294

**Published:** 2025-10-22

**Authors:** Joseph Tooby‐Smith

**Affiliations:** ^1^ Department of Computer Science University of Bath Bath BA2 7AU UK

**Keywords:** interactive theorem provers, physics, proof assistants

## Abstract

Interactive theorem provers (ITPs) are a class of computer programming language that guarantee the correctness of mathematical definitions and theorems written in to them. Within mathematics and artificial intelligence the usage of ITPs is growing. In this perspective, the best usage of ITPs are discussed within another area, physics, and motivate the existence of an open‐source community run project for formalized physics results, PhysLean.

## Graphical abstract

Into an interactive theorem provers (ITPs), one can write mathematical definitions, theorems and proofs, and the correctness of those results is automatically checked. This perspective goes over the best usage of ITPs within physics and motivates the open‐source community run project PhysLean, the aim of which is to be a library for digitalized physics in the ITP Lean.

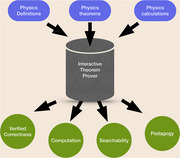

## 1 Introduction

Throughout history physicists have used tools to both do and disseminate their work. These tools have, in some cases, shaped what it means to do physics at research level. An example is the printing press, which in the 1400s led to the dissemination of physics, first through published books, and then in the 1600s through journals. These journals in turn changed what it meant to do physics, introducing the concept of peer‐review.

With the advent of computers more tools appeared. For example in the 1970s Donald Knuth designed TeX allowing authors to typeset their own mathematical manuscripts. This along with another tool, the arXiv, gave us the ability to disseminate new work worldwide overnight. This solidified the concept of preprints within some areas of physics.

Much more recently, the tool of artificial intelligence (AI) models has emerged. This is the first tool at our disposal that has the potential to create new ideas. The field of physics (and academia more generally) is currently undergoing a journey to understand how to best use this tool.

Another tool which emerged with the advent of computers is the interactive theorem prover (ITP). I will give a brief description of an interactive theorem prover here with the details reserved for Section 2. An interactive theorem prover is a computer programming language, like Python, LaTeX, or Microsoft Word, that allows one to write mathematical definitions (or equivalently computer functions), and state and prove theorems about them in such a way that mathematical correctness is guaranteed. The process of writing a definition or theorem into an ITP is often called “formalizing” or “digitalizing” that result.

The fact that correctness is guaranteed is one of the main features that make ITPs so useful to work with. There are other features which include: the ability for structured notation‐independent searches; the ability to make contextual notes; the ability to develop automated theorem proving tactics; the ability to use AI to aid in theorem proving (see e.g., ref. [1]); and the ability to act as a bridge between theory and simulation.

Given what ITPs do, it is not a surprise therefore that over the last decade or so mathematicians and computer scientists have been using this tool. More recently a handful of scientists, including the author have also started to use ITPs.

As with AI, and any new tool, an important question is: how should we best use ITPs in physics in conjunction with the other tools we have available? Naturally, the answer to this question in physics will differ from the same question applied to mathematics, or to computer science. This is because physics is not mathematics, or computer science, and perhaps more importantly the physics community is not the mathematics community, or the computer science community. That said, the answer in physics should be guided from what is being done in these other areas.

In this perspective I will argue for the following main thesis:

**Main Thesis**: The best way to get the most out of interactive theorem provers in physics is to:

1. Have a single open‐source, community run, GitHub repository for formalized physics results, with the following features (Section 4)
  (a) interconnected theories,
  (b) a philosophy of improvement rather than duplication,
  (c) a consistent peer‐review system,
  (d) the ability to have informal results,
  (e) the ability to integrate with knowledge resources.
2. When formalizing results physicists should (Section 5):
  (a) include physics context,
  (b) build APIs based around data structures,
  (c) use AI tools in a controlled manner,
  (d) build physics based automated theorem proving tactics around their data‐structures.

The above thesis suggests an open‐source, community run project, for all formalized physics results. Such a project has been started by the author and is called PhysLean,^[^ ^2^ ^]^ which is based in the ITP Lean 4.^[^ ^3^ ^]^ To prevent repeating the cumbersome wording “open‐source, community run project, for all formalized physics results,” in what follows I will use PhysLean as shorthand. However, note that the principles explored in this perspective extend beyond this project.

I hope that the reader more familiar with the use of ITPs in mathematics will take away from this perspective the differences between the formalization of mathematics and physics, and how these differences might affect its best usage.

The remainder of this perspective will be structured as follows. In Section 2, I will give a general, and high‐level overview of ITPs. In Section 3, I will discuss some of the existing uses of ITPs in physics as an illustration of what can be done. Sections 4 and 5 will argue for my main thesis given above.

## 2 What is an Interactive Theorem Prover?

An interactive theorem prover is a programming language. A comparison between an ITP and Python could be made, since ITPs let you write computer programs and executable functions. A comparison between an ITP and LaTeX or Microsoft Word can also be made, because you can write in an ITP mathematical definitions and theorems, and their proofs. The difference between an ITP and say, LaTeX, is that an ITP checks for mathematical correctness.

There are a number of different ITPs out there, for example Lean, Rocq and Agda, all of which have slightly different features. In what follows I will describe the rough workings of an ITP generically, though throughout this paper we will have a specific focus on Lean.

The basic object in an ITP is a type. A type can be thought of as similar to a set, in the sense that it has elements. In fact, a type can be defined inductively by its elements. For example the type of natural numbers is defined in Lean, and most other ITPs, as


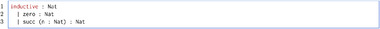


This definition says that Nat has an element zero and for every element n of Nat one has another distinct element succ n . This inductively defines the natural numbers.

Other common types include the type of integers ℤ which is defined similarly to the type of natural numbers, the type of real numbers ℝ , and the type of complex numbers ℂ . There is also the type of types, whose terms are types—this gets into the complex issue of universes, which for the most part can be ignored. In physics one could have for example, the type of electric fields, the type of Lagrangians, the type of fields in a theory etc.

The precise phrase for an element of a type is called a term, and it is usual to write the notation a: T to indicate that a is a term of type T . We say the term a has type T .

From types you can construct other types, for example A → B is the type of functions from the type A to the type B . Another example is the type A × B , which is the type of pairs of objects, one from A and one from B .

Types can carry instances, for example an instance of a vector space, or a ring. The ITP can infer these instances, allowing one to for example, use the notation a + b for any two terms a and b of any type T carrying the instance of a vector space.

A definition in Lean is a specification of a type T and a term of that type. The term might depend on other terms and types. For example, in Lean one can have the following definition


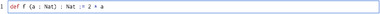


This corresponds to a definition of a term of type Nat based on another term of type Nat. This is actually equivalent to defining a term of type Nat → Nat as follows


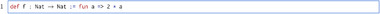


Despite being a mathematical definition within an ITP, this function would also be executable allowing, e.g., f 2 to be calculated automatically.

In Lean one can also define objects through structure . A structure has multiple fields which can be of different types, for example, the product of two types A × B can be defined through structure with one field of type A and another of type B . We will see an explicit example of a structure in the next subsection. A similar construction to structure appears in other ITPs.

An ITP knows the type of everything, and this is basically how it carries out its mathematical correctness checks. It checks that the types of terms are correct, given the context in which they appear.

Now let us turn to theorems. The statement of a theorem is made by specifying a special class of Types called Prop. A proof of a theorem corresponds to specifying a term of the corresponding type. The key difference between a theorem and a definition (which is really down to a slight difference between a Prop and a Type ) is that for a theorem the term itself does not matter whilst it does for a definition. An example of a Prop is (propositional) equality, defined in Lean as


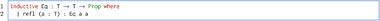


In some ITPs there are tactics that help you in specifying terms of Props, i.e. prove theorems. These tactics manipulate the types present by applying previously known theorems and results. Importantly, by using tactics it is possible to work using high level mathematics, without much consideration of the underlying type theory present. This makes proving results in an ITP not too dissimilar to proving them on pen and paper.

It is worth noting here what makes ITPs different from one another. This is usually down to the underlying type theory used, and additional axioms introduced.

## 3 Use of Interactive Theorem Provers in Physics

In this section we discuss the use and in more detail the benefits of formalizating physics into Lean.

### 3.1 Existing Formalizations

To give the reader an idea of what can be done within an ITP in physics, in this section I will give a brief review of some of the literature.

First, I will detail some stand‐alone publications followed by an account of areas formalized within the PhysLean project.

Within classical mechanics, there has been an attempt to formalize Lagrangian mechanics in Isabelle/HOL within the thesis.^[^ ^4^ ^]^

There have been two stand‐alone publications on the formalization of results from Optics. The first ref. [5], in the ITP HOL Light, covered parts of optical systems. The second is a Bachelor's thesis^[^ ^6^ ^]^ which covered aspects of quantum optics.

There are a number of stand‐alone projects formalizing parts or aspects of special relativity. These include ref. [7] in Rocq and refs. [8, 9, 10] in Isabelle's archive of formal proofs.

The group of Tyler Josephson have been working in physical–chemistry, and have formalized aspects of thermodynamics in Ref. [11], and Lennard Jones energy calculations in ref.  [12]. The focus of these papers is in the direction of verified simulations.

Turning to formalizations currently within PhysLean, there is currently coverage in the following areas: electromagnetism, condensed matter physics, statistical mechanics, quantum mechanics, particle physics and string theory. Some examples of explicit results formalized include:

1. Wick's theorem in particle physics,^[^ ^13^ ^]^
2. the quantum harmonic oscillator in quantum mechanics,
3. Maxwell's equations in terms of functions and distributions,
4. properties of the tight binding model in condensed matter physics,
5. the electrostatic properties of a point charged particle in 1 dimension and 3 dimensions,
6. variational calculus and the proof of the Euler–Lagrange equations,
7. the canonical ensemble,
8. properties of the Higgs potential,
9. index notation for representations of the Lorentz group including fermions,^[^ ^14^ ^]^
10. etc.

To give an explicit example of a result formalized in PhysLean in the code block below, I give part of the file on the formalization of the Higgs potential. I do not expect the reader to understand all of the details here as it is out of the scope of this perspective and is merely provided as an illustration.


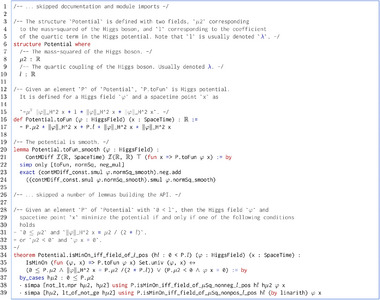


The result Potential is the input data of the Higgs potential, that is the coefficient of the mass term μ2 and the quartic term *l* (one can't use λ in Lean as this has a special meaning in type theory). The result Potential.toFun is the potential, which takes as an input a Higgs field and a point in space‐time. The results Potential.toFun_smooth , which proves the smoothness of the Higgs potential, and Potential.isMinOn_iff_field_of_*l*_pos which proves the minima structure, make up the API around the potential for the Higgs field (see 5.2).

It is noted that there are obvious improvements which could be made to this formalization by e.g. deriving the potential from an algebra consisting of the Higgs field, and invariance under the standard model gauge group (see 4.2 for a discussion around improvements). We invite the reader to view the PhysLean repository for more examples of formalizations.

The main point I would like the reader to take away from this subsection, is that one can use ITPs to do high‐level standard physics, and their use is not restricted to special areas of mathematics or for example, axiomizations. We will discuss what can and cannot be formalized in more detail in the next subsection.

### 3.2 What Can and Cannot Be Formalized?

In the above section we detailed results from physics which have already been formalized in an interactive theorem prover. I now wish to discuss more generally what results from physics can likely be formalized.

It is well known that different areas of physics require and depend on different levels of mathematical rigor in their work. For example, in mathematical physics this rigor is often absolute, whilst as one gets closer to experiments the rigor required often decreases.

The real determining factor of whether something in physics can be formalized or not is whether it permits a mathematically well‐defined formulation. It is important to understand what this means, and to do this I will illustrate with a number of examples.

The first example is setting a term in a Lagrangian to zero, as is often done when making approximations to make problems more tractable. In the above code snippet of the Higgs field one could set the μ2 in the potential equal to zero by adding a hypothesis h: μ2 = θ in the desired places. Such an approximation does not have a mathematical rigorous justification, but it does have a mathematically well‐defined formulation, and therefore can easily be formalized in Lean.

The second example is the path integral used in quantum field theory. It is well‐known that the path integral does not have a mathematically rigorous formulation. However, the process one undertakes to go from a lagrangian to a scattering amplitude does have a mathematically well‐defined formulation. In particular, the steps the physicist takes are all mathematically well‐defined, even though they don't have a mathematically rigorous justification.

A third and final example is one which would lead to difficulties in formalizations, which may or may not be surmountable. This example is related to Sorites paradox and is down to the use, and definition of approximations. Physicists may approximate both 800 and 1200 by 1000 in an expression, but may not be comfortable approximating 1200 by 800. Because of this it is difficult to give such an approximation a mathematically well‐defined formulation, and therefore formulate it into Lean. However, there are many areas and results of physics which do not rely on such types of approximations.

It is worth adding to the above discussion that a lot of what can be formalized depends on a good choice of starting point. Choosing a good starting point to a formalization can make it tractable, yet the formalization still useful. The most natural, and arguably best starting point for any formalization is an axiomatization of the underlying area. However, restricting to such starting points greatly limits the formalizations one can carry out right now, and postpones the other benefits (discussed in the next subsection) one can get from formalizing physics.

Any formalization that one does in an ITP will be mathematically correct. However, it is important to note that an ITP does not prevent you from using a non‐physically‐meaningful starting point of your formalization, the wrong definition of a key component, or adding assumptions which contradict one another. Also, it does not prevent you from proving results based on a said starting‐point, key component or assumptions. Indeed, from contradicting assumptions one can prove anything. This is true even in mathematics, not just physics. Thus, with any formalization, it is important that one checks the starting point, the definitions made, and the assumptions used, before drawing a conclusion about the wider correctness of a result.

A topic one may also worry about is the use of computational techniques in physics to “prove theorems,” e.g., with Mathematica or other computer algebra systems. It will be the subject of a future paper to show that such computational proofs can often be formalized in Lean with care, and to give an explicit example of this.

### 3.3 Benefits of Formalization Physics

In the introduction to this paper I briefly mentioned some of the benefits one can gain by formalizing results from physics into Lean (see also ref. [15]). In this subsection I will expand on this by focusing on the question: how can Lean fit into the physicist's workflow? We will see more examples of this in the sections.

First and foremost, physicists can use Lean to mathematically check their own results, and see explicitly how it connects to other results in the literature. They can also use it to determine precisely what assumptions they have used and what are actually needed.

Second, they can use Lean to search for results which may be needed in their own proof, or to learn about the existences of new results. Tools such as Loogle^[^ ^16^ ^]^ allow one to make a search based on mathematical structure not just text. For example searching Loogle for “HiggsField” will pull up any result dependent on this type, which may not necessarily be the case from a text‐based search.

Third, they can use AI to help prove theorems. AI models have been increasingly used in Lean to help “theorem prove.” The main advantage of this is that the human, and AI model, can be completely sure of the mathematical (not physical) correctness of the results produced by the AI. I will discuss this more in Section 5.3.

Fourth, because Lean is a fully fledged programming language, one can write simulations and programs directly in Lean. These can use the exact same definitions which are used to prove proper mathematical theorems. Thus, you can talk about theory and simulation in the same consistent framework.

Last, with careful planning and structuring, one can use Lean as a pedagogical tool to help teach Lean, or physics. This opens a whole new possibility for students and researchers to learn new subjects.

It is important to make a brief remark on the difference between Lean and computer algebra systems. Computer algebra systems, like Mathematica, are very good at performing calculations, algebraic and symbolic manipulations, but one cannot prove theorems or define every mathematical structure in a computer algebra system. One can do these things in ITPs, and work is being done to improve the “computer algebra” nature of ITPs.

## 4 Arguing for PhysLean

The key component of the thesis of this perspective is the project PhysLean.

The inspiration for such a project comes from open source projects in computer science and the project Mathlib. Mathlib is an open‐source community‐run project to formalize results from mathematics into the interactive theorem prover Lean. However, it is not the case that all formalized results from mathematics end up in Mathlib. This is in part due to the use of different ITPs within some communities of mathematicians. For example mathematicians working on homotopy type theory, or adjacent areas, are naturally going to use an ITP which is based on this specific type theory, which Lean, for example, isn't. Thus, the question arises, does using one ITP throughout physics suffice? In my opinion the answer to this question is yes, for the simple reason that physicists rarely rely on areas of Mathematics like homotopy type theory where a different use would be justified. Therefore, I feel that a consistent ITP can, and should, be chosen in all areas of physics, and due to the presence of Mathlib with its focus on classical mathematics, it seems natural that the ITP used should be Lean.

One may wonder at this point why we need PhysLean if we already have Mathlib, and could the results from PhysLean not go into Mathlib? I believe the answer to this question is no, for the following reason. In physics one cares about the physics context and mathematical context of a result. Compare this to mathematics, where one only cares about the mathematical context of a result. Including this physics context into a formalization (see Section 5.1), to give the reader the underlying physics story is important, and would not be applicable in Mathlib. There is also the fact that Mathlib requires a level of generality of results which is not applicable to all physics, for example the explicit study of specific matrices relevant to physics, such as the Cabibbo–Kobayashi–Maskawa matrix in particle physics. In short, Mathlib and PhysLean serve different fields and different community needs.

Before arguing why I think the project PhysLean should exist, let me address two possible downsides to having such a project, both of which are due to cultural aspects.

The first downside is the following. Physicists (except experimental high energy physicists) are used to working in small teams or as individuals, often in secret, and then publishing papers based on that work. Having an open source project like PhysLean would require a more collaborative approach with, in some cases, work added to the project piecemeal. I believe the necessary cultural shift can be undertaken to permit such collaborative work, whilst also keeping the authors abilities to write individual papers. This requires some sort of consensus to be formed within the community.

The second counterargument to having PhysLean is down to different cultures between different areas of physics, and how they may conflict with each other when working on the same project. I think we can optimistically learn from the mathematicians here, that these potential conflicts is something which can be overcome. One potential way to help prevent such conflicts is by having clear policies and principles which the project follows.

Let me now turn to the argument for having PhysLean. The main arguments will be contained within the following subsections where I will discuss the proposed features of such a repository. Most of these features are only possible with the existence of PhysLean while others would be extremely onerous to carry out without PhysLean.

Before describing these features let me give one additional argument for PhysLean. PhysLean is a synergy of two tools, ITPs and GitHub. GitHub is usually the purview of the computer scientists, and we can learn from their use of it. Their usage is typically to have big open‐source community run projects or libraries (think of the Python package “SciPy” for example). There are three main reasons for this. First, it keeps all similar results up‐to‐date with each other, using the same version of dependencies. Secondly, it makes it easier for the user, who only has to find, download, and learn to work with one library. Lastly it allows for standardization in for example, naming, layout etc., which can be enforced with automated scripts called GitHub workflows. All of these reasons extend both to Mathlib, and PhysLean and therefore motivate the existence and need for the latter.

I will now describe and argue for the main features of PhysLean stated in the main thesis of this perspective.

### 4.1 Interconnected Theories

There are two possible ways one could organize PhysLean. The first is to have each physical theory as a stand‐alone formalization, built independently of all other theories. The second is to have each theory connected to one another, for example, by depending on the same definitions, and/or with appropriate theorems.

To illustrate these two approaches in more detail, let us give an example using electromagnetism. There are two theories of electromagnetism, one using standard vector calculus and the other arising from gauge field theory. In the first method of organization these two theories would be developed independently of one another. In the second approach, they would be connected, for example, with a theorem that connects the field strength arising from the gauge field theory to the vector calculus definition of the electric and magnetic field.

I believe that the second approach is the one which should be followed. However, it is worth considering two advantages that the first approach may have over the second. To illustrate these two advantages, we continue to use the example of electromagnetism. The first advantage is the lightweight nature of the program. If I wanted to use the vector calculus version of electromagnetism in the first approach this is all my computer would have to build, but in the second approach, it would have to build the whole of gauge field theory, which can be both time and memory consuming. The second advantage is related to ease of use. Most undergraduates know the vector calculus approach to electromagnetism, but few know the gauge field theory approach. In the first approach there is no way that the undergraduate would get confused or stuck by something in gauge field theory. This is possible in the second approach.

I state these two potential advantages of the first approach, because, if when following the second approach one thinks and understands these downsides, they can be mitigated by careful design and organization of the formalization.

Aside from the obvious benefit the second approach has over the first — which is that one can more easily move between theories and see their connections — there is also a less obvious one related to axiomatization. In general, formalization of physics does not imply axiomatization, although it is one area where formalizing can be useful. Let us use special relativity as an example, and suppose we have two axiomatizations A1 and A2 of special relativity, as well as the core undergraduate taught theory C. Following the first approach above, one would formalize A1, A2 and C separately, whilst in the second approach one would be able to connect both A1 to C and A2 to C, and also investigate possible connections between A1 and A2 themselves. In an ideal situation you would not want C tied to one or other axiomatization, and this again requires careful design, especially if neither are considered standard.

### 4.2 Improve Upon Not Duplicate

Consider the following situation. Alice is formalizing a physical theory P. The theory P depends on some foundational material F, which has previously been formalized by Bob. But Bob's formalization of F does not meet Alice's needs.

What are Alice's options? Her first option is to completely reformalize F in a way which does meet her needs. This sort of behavior is followed in academic papers, where explanations of the same concept are repeated in lots of different papers, each fitting the differing contexts and the idiosyncrasies of the authors.

Alice's second option is to modify or improve Bob's formulation of F so that it does meet her needs. This is the approach taken with Wikipedia, or the nlab, as well as with open source computer science projects.

The author believes that when formalizing physics one should have the philosophical view that the second approach is better due to two main advantages. The first is that it forces improvements to be simultaneously propagated through the whole library. This has the added benefit of ensuring and preserving compatibility between different areas of the library, which is related to the previous section on interconnected theories.

The second advantage is that a third user, Charlie, does not have to make a choice between different formalizations of F, as they would if the first approach was followed. This leads to a better, and more consistent user experience.

The second approach however without its downsides. The first is the added work Alice would need to do to both improve F and modify existing formalizations dependent on F, compared to just redoing F for her own needs. There are two comforting remarks that can be given to Alice in this situation. The first is that others will have gone through the same pain, and she is benefiting from their work. The second is that the process of having to adjust all the results that depend on F with her improvements or changes would allow Alice to be sure she is not breaking any of the functionality of Bob's formalization. This thereby acts as a litmus test for Alice's changes.

The second downside to this approach is the following. Suppose Bob has formalized F, and is writing a paper on this formalization or related results, and Alice comes along and improves or modifies the formalization of F. The downside is the dilemma Bob now faces. Does Bob include Alice on his paper, and/or talk about Alice's formalization instead of his own? I believe that the solution to such questions can be made on a case‐by‐case basis, as they are currently on a more local scale. But I also believe that some community guidelines could be created to potentially ease the worries of some authors who may want to formalize results, but may be put off by such dilemmas.

A third downside, is related to whether Bob agrees with or consents to Alice's supposed improvements. Perhaps what Alice thinks is an improvement is, in the eyes of Bob, not. A solution to this problem is the subject of the next subsection.

### 4.3 Transparent Peer‐Review

Suppose Alice, from the previous subsection, improves and makes changes to Bob's formalization of F. Who decides if Alice's improvements are good enough to make it into PhysLean or not?

There are two complementary ways of doing this. The first is to use automated computer checks to ensure Alice's work meets certain standards. The ITP itself will check for mathematical correctness, so these additional checks correspond to things like: ensuring all definitions have documentation, not having unwanted double spaces, and ensuring correct indentation. Via the synergy of ITPs with GitHub in PhysLean such automated checks are easy to implement using GitHub workflows, as mentioned above.

The second way to ensure Alice's improvements are good enough is via a peer‐review process. The idea is the following. Suppose Alice submits her changes to F to the project, another expert Emma, say, in F would look at Alice's changes and determine their quality and if they are actual improvements. Emma would then give her advice to a team of maintainers about whether the improvements should be merged. The maintainers, like an editor of a journal, would follow that decision in all but the extreme cases.

This is the exact process that happens in Mathlib and countless other open‐source projects, and it is again easily implemented via GitHub.

I advocate for both these mechanisms to be present, as they not only maintain the project's quality, but they also act to prevent idiosyncrasies from emerging in different modules. Prevention of idiosyncrasies has the added benefit of increasing ease of user experience.

Exploring the peer‐review process further, I believe there are two important features it should have. The first is speed. The changes Alice made to F may only be very small, and Alice, and others may be dependent on those changes for future work. If the review process takes too long, this may (depending on Alice's workflow), stagnate her progress, or worse still she may lose interest in formalization entirely, due to the burden of the peer‐review process.

The second feature is transparency. Transparency is important, as it not only aids to speed up the review process, but it ensures a level of fairness between contributors. In particular a contribution to PhysLean should be judged as systematically as possible on its content, not on who wrote it.

### 4.4 Informal Results

Suppose Alice is now a physicist who is an expert in area E, but who, like most physicists, does not know how to use an ITP. I believe it should be possible for Alice to still contribute to the digitalization of physics using her expertise in E.

This can be facilitated in a project like PhysLean using the notion of informal results. Informal results are placed as temporary substitutes (perhaps long term) for formal results within the project. An example of an informal result in PhysLean is:


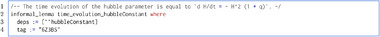


Here, the first line is an English statement of the result; line 2 contains the name of the result; line 3 the dependencies of the result; and line 4 a unique identifying tag which is used to produce a list of all informal results.

The idea is that Alice would be able to write these informal results based on her expertise in E in sufficient detail that for example Bob, who knows how to formalize results but may be less familiar with E, can turn Alice's informal results into formal results.

A similar concept has been used in the mathematics community in the form of Lean blueprints.^[^ ^17^ ^]^ These are latex generated documents which guide the formalization of results. An example of a project which is currently ongoing and using blueprints is the Fermat's last theorem project.^[^ ^18^ ^]^

The advantage of this workflow is that it can put to best use the expertise of individuals in the most efficient way. For example, there is no point in Alice trying to formalize something when this does not meet her personal skill set.

Having a project like PhysLean allows the necessary infrastructure to be built around these informal results, making them as easy to find, and formalize as possible.

### 4.5 Integration Into Knowledge Resources

Historically, academic knowledge has sat within academic papers. With the advent of AI, and its heavy reliance on data sets, there has been a proposal for a multipurpose knowledge resource in ref. [19].

As mentioned in that paper, Lean and ITPs can provide a well‐founded way in which some human knowledge can be stored.

An open source project like PhysLean can be seen as a store or as a supply of such knowledge. Furthermore, the necessary infrastructure and standards could be built into PhysLean to make interactions with a knowledge resource as efficient and effective as possible. This will require engagement and collaboration between and across the communities. When the foundations of such a knowledge resource emerge I advocate for such a collaboration.

## 5 Motivating Aspects of General Usage

Beyond the argument for a single open‐source repository, PhysLean, the thesis of this paper includes more general recommendations on how physicists can most effectively use interactive theorem provers. In this section I give the arguments for each of these recommendations.

### 5.1 Include Physics Context

A definition or theorem in physics contains additional physical context beyond its mathematical statement. For example, it is possible to have two theorems or two definitions in physics which are mathematically the same statement, but which carry different physical context.

An explicit example is the Hilbert space of a qubit and the target space of the Higgs field. Mathematically both these correspond to the vector space ℂ^2^ , but they correspond to physically different objects, i.e., when the physical context is included.

When formalizing physics, I believe it is important to include this physics context within the formalization to help the reader understand what is being formalized and why.

There are a number of ways that physics context can be included in a formalization of an area of physics. The first is by ensuring the name of the definition or theorem matches the physics underlying it. For example, the name of the Hilbert space of a qubit could be qubitHilbertSpace whilst that of the target space of the Higgs field could be targetHiggsField . Some ITPs will allow you to cast from qubitHilbertSpace to targetHiggsField , i.e., to treat an element of the former as an element of the latter. In a lot of cases, this does not make physical sense, and certain tricks can be played to prevent such casting and in doing so prevents certain “physics” mistakes being made.

The second way to include physics context is via documentation. Like most programming languages, ITPs allow you to include documentation surrounding and connected to different parts of the code. For example in Lean, one can give files a designated documentation string which is usually used to explain their content. One can also give documentation strings to individual theorems and definitions. These documentation strings are usually used to give an English written description of the result.

Such documentation can be, and I believe should be, used to include the physics context of the underlying result. Not only does this have an advantage when it comes to a user reading and understanding a formalization, but it also makes it easier to search for results both with algorithmic methods, e.g., Loogle^[^ ^16^ ^]^ or AI methods, e.g., Lean Explore.^[^ ^20^ ^]^ These documentation strings can be supplemented with references to the literature, connecting the formalized result to their informal result, and related discussions.

The last way to include the physics context within a formalization that I will discuss, is through organization. Results related to the same area of physics or the same physical object can be placed together in the project. This should surpass an organization based on the mathematical context of the results, as is done in say, Mathlib. Such an organization can make it clear what area of physics a formalized result is related to, and what it is trying to achieve. This is related to the content of the next subsection.

### 5.2 Build APIs Around Data Structures

Computer scientists are in general, interested in data structures, for example, lists, arrays, and dictionaries. Thus, when building a library of results they will often first identify the key data structures present, and then for each of these data structures create an API, i.e., lots of operations or functions on those data structures. These operations may not be of immediate use to the particular computer scientist, but may be of use to others in the future.

This also influences the organization of the library that they build. For example, a computer scientist might identify a list as a key data structure for their library. Thus, they may create a file which starts with the definition of a list and then defines sorting of lists, reversing of elements, inserting elements etc. In other words, define or create an API around lists. Another data structure of interest to the computer scientist might go into a different file.

This holistic approach of the computer scientists should be compared with the approach taken by physicists when writing a paper. Typically, physicists will identify the key theorem or result they want to prove and give just enough definitions and surrounding context to ensure that the key result, and perhaps it's proof, makes sense. This is, in a sense, the opposite point of view to the computer scientist.

It is not surprising that taking the computer scientist's approach is in the long run, more useful when formalizing physics than taking the approach traditionally followed by physicists. The main reasons for this is that it ensures that results are present for future users, and the building of an API acts as a test for the practicality of a given definition.

To explore how this works in practice, let us give two examples from PhysLean. The first example is from quantum mechanics (QM). For the 1d harmonic oscillator in QM, there are a number of data structures involved: the Hilbert space; the collection of physical parameters; and the set of solutions. In PhysLean, each of these data structures has its own file and API built around it.

The second example is Wick's theorem from quantum field theory, the formalization of which was presented in ref. [13]. Here, there are a number of key data structures, the most important of which is Wick contractions. A lot of the formalization of Wick's theorem in PhysLean is down to building a large API around these Wick contractions, and this API sits within a clear and distinct file system within the project.

The approach outlined here is the approach taken in Mathlib. However, I wish to highlight one important consideration that physicists should take into account. In some ITPs, Lean being one of them, it is possible to create definitions which are not computable, i.e., cannot be put or used in a computer program which can be executed. This is often due to the presence of an axiom of choice. Mathematicians rarely care about non‐computability, but physicists often want to make programs with their definitions. As such I believe it is important, where possible, to ensure that the data structures the APIs are built around are computable.

### 5.3 Controlled Synergy with AI

Similar to how AI can be used to write code into Python, an AI can be used to help write formalizations into an ITP. In some cases the correctness or incorrectness of the formalization can be fed back into the AI, and the AI can adjust its formalization accordingly. This automatic feedback loop is why some companies, like Google DeepMind and Harmonic, are turning to Lean to help their AIs reason.

In this section I want to focus more generally on the use of AI in helping formalize physics, whether that is by simply using an LLM like ChatGPT or Lean specific programs like LeanCopilot^[^ ^21^ ^]^ which take advantage of the aforementioned feedback loop.

I wish to argue that whenever an AI formalizes physics, a human should always act as a gatekeeper. This is not least because we should be formalizing physics for humans, and as such a human should be in charge of deeming whether a formalization is useful to us.

More practically though, even with the above feedback loop, there is plenty of room for AIs to hallucinate when formalizing physics. This is perhaps more of an issue in physics than in mathematics, due to the additional physics context around the definitions and theorems that must be present. But the AIs can also hallucinate in the definitions themselves, and if they do, the theorems that they end up proving may be theorems about the wrong things. The definitions the AI makes may be practical only for the given task the AI has been given and not more widely applicable.

These hallucinations if not kept in check can serve to dilute the information quality of formalized physics, and it could, eventually get to the point where it becomes unusable. Similar arguments can be applied in other programming languages and libraries, and more generally to the internet.

### 5.4 Physics Based Theorem Proving Tactics

One of the key advantages of an ITP like Lean is that their tactics can help with proofs. These tactics, often written in a meta‐language, make ITPs easier and more enjoyable to work with. However, the tactics do not come free, they have to be developed and made usable.

To give an example of a tactic, Lean has the tactic ring . The idea of this is to prove relations which hold due to the basic properties of a ring, e.g., commutativity and associativity of addition, and distributivity of multiplication over addition. For example if a b c: A where A carries the instance of a ring, then the tactic ring will be able to solve a * (b + c) = c * a + b * a .

There are data structures of particular interest to physicists, for example, Feynman diagrams, Lagrangians, gauge fields etc. Some of which have automated tools to use and manipulate them in other languages like Mathematica.

I argue that effort should be put into developing such tools in Lean. More generally, I argue that whenever a physicist makes a data structure, they should ask themselves if there are any tactics which would make working with that data structure easier. In some cases, the time and effort needed to make such tactics will not be worthwhile, and in others, it might constitute its own research project, but in general there should be a research effort in developing these tactics.

## Conflict of Interest

The authors declare no conflict of interest.
